# Sex differences in distribution of cannabinoid receptors (CB1 and CB2), S100A6 and CacyBP/SIP in human ageing hearts

**DOI:** 10.1186/s13293-018-0209-3

**Published:** 2018-11-27

**Authors:** Żaneta Piotrowska, Michał Niezgoda, Wojciech Łebkowski, Anna Filipek, Natalia Domian, Irena Kasacka

**Affiliations:** 1 Department of Histology and Cytophysiology, Medical University of Białystok, Białystok, Poland; 2 Department of Neurosurgery, Medical University of Białystok, Białystok, Poland; 3 Nencki Institute of Experimental Biology, Polish Academy of Sciences, Warsaw, Poland

**Keywords:** CB1, CB2, S100A6, CacyBP/SIP, Human, Heart, Sex differences

## Abstract

**Background:**

Women live about 4 years longer due to lower prevalence of cardiovascular complication with ageing. However, the mechanisms involved in the preservation of heart functionality in women have not been fully elucidated.

The endocannabinoid system fulfils a significant role in the regulation of cardiovascular system functioning. Cannabinoids, acting through specific receptors (CB1 and CB2), influence on blood pressure, heart rate and myocardial contractility. The function of cardiac muscle cells is strictly dependent on calcium ions. Calcium homeostasis in cardiomyocytes is subjected to complex regulation via calcium-binding proteins. Among them, increasing attention has been paid to the recently discovered S100A6 and CacyBP/SIP.

In order to better understand sex differences in the regulation of cardiomyocyte function during ageing, we undertook the present research aimed at immunohistochemical identification and comparative evaluation of cannabinoid receptors, S100A6 and CacyBP/SIP, in the myocardium of ageing men and women.

**Methods:**

The study was conducted on the hearts of 12 men and 10 women (organ donors) without a history of cardiovascular disease. The subjects were divided into two age groups: subjects older than 50 years and subjects under 50 years old. Paraffin heart sections were processed by immunohistochemistry for detection of cannabinoids receptors, S100A6 and CacyBP/SIP. In the heart samples from each study, participant’s expression of genes coding for CB1, CB2, S100A6 and CacyBP/SIP using real-time PCR method was measured.

**Results:**

CB1 and CB2 immunoreactivity in the cytoplasm of cardiomyocytes in the heart of subjects over 50 was weaker than in younger individuals. In the heart of younger men, CB1-immunoreactivity was weaker and CB2-immunoreaction was stronger compared to women. In the hearts of older men, the CB1-immunostaining was more intense and CB2-immunoreactivity was weaker than in women. Immunodetection of CB1 shoved the presence of receptor in the intercalated discs, but only in the hearts of individuals over the 50 years old. In the hearts of older individuals, stronger immunolabelling was observed for S100A6 and CacyBP/SIP. Male hearts had greater S100A6-immunoreactivity (both age groups) but less CacyBP/SIP immunostaining (individuals over 50 years) compared to the age-matched women. The expression of genes coding CB1, CB2, S100A6 and CacyBP/SIP in the human heart was sex and age-dependent. Observed changes between men and women as well as between subject under and over 50 years were consistent with immunohistochemically stated changes in peptide content.

**Conclusion:**

Together, the data presented here indicate a close interaction between ageing and sex on the distribution and levels of cannabinoid receptors (CB1, CB2), S100A6 and CacyBP/SIP in the human heart.

## Background

Increased interest has been expressed in recent literature in the biological action and potential medical application of compounds contained in *Cannabis sativa* plants or their synthetic analogues [1]. Cannabinoids, acting through specific receptors called CB1 and CB2, modulate various physiological and pathological processes within the body. Cannabinoids impact mental health, eating behaviour, reproductive function, pain sensation and immune response [1]. The latest reports also underline the importance of cannabinoids in the control of cardiovascular system function.

Cannabinoids induce hypotensive and bradycardic effects as well as influence heart contractile action [1]. Stimulation of the CB1 receptor results in the weakening of cardiomyocyte contractility, while activation of the CB2 receptor increases the force of cardiac muscle cells contraction [2]. A number of experimental data have revealed a significant impact of cannabinoids on cardiomyocyte survivability and their involvement in histopathological changes in the heart [3–11]. Research on rodent models of cardiomyopathy has demonstrated that the activation of the CB1 receptor triggers cardiomyocyte injury, augments collagen deposition and cardiomyocyte overgrowth whereas CB2 receptor pathways evoke cardioprotective, antifibrotic and antihypertrophic action [3–11].

Cannabinoids regulate intracellular calcium content in cardiomyocytes, which is crucial for cardiac cell contraction and relaxation [1, 12]. Cannabinoids modulate calcium ion current through membrane channels (L-type Ca^2+^ channels, T-type Ca^2+^ channels, Na^+^/Ca^2+^exchanger) and calcium oscillations between the sarcoplasmic reticulum and the cytosol [1, 12].

Calcium homeostasis in cardiac muscle is subjected to complex regulation on the part of calcium-binding proteins. The S100 protein family constitutes the largest group of proteins capable of binding calcium ions [13]. The S100 protein family includes more than twenty low molecular weight proteins with two EF-hands Ca^2+^-binding motifs [13]. A growing body of evidence points to the significance of S100A6, one of the recently identified S100 proteins, in cardiovascular system performance [14–17]. The S100A6 potentiates Ca^2+^ release from sarcoplasmic reticulum stores during the contraction cycle, which implies that it may influence cardiomyocyte contractility [15]. The in vitro and in vivo studies have shown the beneficial effect of S100A6 on cardiomyocyte viability [15, 16]. The protein reduces apoptosis of cultured cardiomyocytes subjected to hypoxia and tumour necrosis factor-alpha (TNF-α) and prevents the injury of cardiac muscle cells in mice after myocardial infarction (MI) [15, 16]. The latest reports also demonstrate the protective properties of S100A6 towards hypertrophic and fibrotic changes of the heart wall [15, 17].

The S100A6 protein interacts with various intracellular molecules and modulates their activity in a calcium-dependent manner [14]. One of the most important targets of S100A6 is calcyclin-binding protein/Siah-1-interacting protein (CacyBP/SIP) [18, 19]. The CacyBP/SIP protein is responsible for the coordination of a great variety of cellular processes including the proteosomal degradation of proteins, cytoskeletal organisation, regulation of gene expression, cell proliferation and differentiation [18]. There are few reports concerning the influence of CacyBP/SIP on cardiovascular system function, and therefore, the knowledge in this area is fragmentary [20]. Studies on neonatal rats and cultured cardiac myoblasts (H9C2 cells) have demonstrated the important role of CacyBP/SIP in heart development, since the protein was found to promote cardiomyocyte differentiation and myotube formation [20]. The same investigation revealed that CacyBP/SIP attenuates cardiomyocyte damage in conditions of hypoxia-reoxygenation stress [20].

Epidemiological data indicate a higher prevalence of cardiovascular disease and increased cardiovascular mortality in men in comparison to women [21, 22]. Heart complications appear at an earlier age and progress more rapidly in men compared to women [21, 22]. In men, ageing associated with gradual cardiac muscle cell loss and hypertrophy of the remaining cardiomyocytes [23], while in women, the total number of myocardial cells remains relatively constant during their life span [23].

However, the mechanism/mechanisms responsible for the improved preservation of heart structure in older women have not been fully elucidated.

In order to better understand sex differences in the regulation of cardiomyocyte function during ageing and considering the aforementioned importance of the cannabinoid system, S100A6 and CacyBP/SIP in cardiac function control, we decided to performan evaluation of cannabinoid receptors, S100A6 and CacyBP/SIP in the myocardium of ageing men and women.

The aim of the present study was the immunohistochemical detection and comparative assessment of cannabinoid receptors (CB1 and CB2), S100A6 and CacyBP/SIP in the heart of men and women in different age groups.

Considering the significant changes in heart homeostasis during ageing, the disturbance in cannabinoid receptors, S100A6 and CacyBP/SIP in the heart of older patients might be expected. Bearing in mind more effective preservation of heart structure in elderly women compared to men, age-related changes in cannabinoid receptors, S100A6 and CacyBP/SIP in the heart might have different expression in both sexes.

## Methods

### Sample collection

Twenty-two adult subjects (12 men and 10 women) without a history of cardiovascular disease were used in the study. The subjects were in the age range from 19 to 65 years, mean body weight 75.5 ± 2.24 kg, and mean BMI (body mass index) 25.0 ± 0.52 kg/m^2^. The men and women were divided into two groups: subjects older than 50 years and subjects under 50 years old (Table 1).

**Table 1:** Average age [years], body weight [kg] and BMI [kg/m^2^] in studied men and women

Group of patients		No. of patients	Age (years)	Weight (kg)	BMI (kg/m^2^)
Under 50 years old	Overall	11	35.0 ± 2.58	74.8 ± 3.14	24.7 ± 0.65
	Men	6	37.9 ± 2.96	85.1 ± 1.97	26.4 ± 0.43
	Women	5	31.4 ± 4.36	61.9 ± 2.21 ^♯^	22.6 ± 0.93 ^♯^
Over 50 years old	Overall	11	57.8 ± 1.35*	76.6 ± 3.16	25.4 ± 0.91
	Men	6	58.4 ± 1.90 *	83.7 ± 1.32	26.2 ± 0.34
	Women	5	57.0 ± 2.04 *	66.6 ± 4.44 ^♯^	24.5 ± 2.17 ^♯^

Data present mean ± SE

*p < 0.05 patients over 50 vs patients under 50 years old

^♯^*p* < 0.05 women vs men belonging to the same age group

Each study participant presented with clinical symptoms of brain death was considered to be an organ donor. Irreversible brain damage was confirmed by special clinical examination and angiography (no blood flow within the brain arteries). After brain death was diagnosed and confirmed, from each body, organ tissues were harvested for transplantation (kidneys, liver and heart). Immediately after the donor’s heart was excised for transplantation, samples of the heart tissue from each body were collected.

Heart samples were immediately fixed in Bouin’s solution and routinely embedded in paraffin. Sections (4 μm) were stained with haematoxylin-eosin for general histological examination and processed by immunohistochemistry for the detection of cannabinoids receptors (CB1 and CB2), S100A6 and CacyBP/SIP.

### Ethical issues

The study protocol was approved by the Ethics Committee, Medical University of Białystok (R-I-002/345/2007), and a written informed consent had previously been obtained from each subject or from his/her family member (s).

### Immunohistochemistry

Paraffin blocks were cut into 4-μm sections (three sections from each subject for each antibody) and attached to positively charged glass slides. Immunohistochemistry was performed, using an EnVision Plus-HRP Rabbit Detection Kit K4011 (Dako Denmark) [24]. Immunostaining was performed by the following protocol: paraffin-embedded sections were deparaffinised and hydrated in pure alcohols. For antigen retrieval, the sections were subjected to pre-treatment in a pressure chamber heated for 1 min at 21 psi at 125 °C (one pound-force per square inch [1 psi] equates to 6.895 kPa, the conversion factor has been provided by the United Kingdom National Physical Laboratory). During antigen retrieval, sections were incubated with Target Retrieval Solution with pH 9.0 (S 2367, Dako Denmark) for CB1 or Target Retrieval Solution Citrate pH = 6.0 S 2369 (Dako Denmark) for CB2, S100A6 and CacyBP/SIP. After cooling down to room temperature, the sections were incubated with Peroxidase Blocking Reagent S 2001 (Dako, Denmark) for 10 min to block endogenous peroxidase activity. Subsequently, the sections were incubated overnight with the primary antibodies against CB1 (No ab23703 purchased from Abcam, UK), CB2 (No ab3561 purchased from Abcam, UK), S100A6 and CacyBP/SIP (both purchased from Nencki Institute of Experimental Biology, produced in-house as described in a report by Kuźnicki et al. [25] and Jastrzębska et al. [26]), at 4 °C in a humidified chamber.

The antisera were previously diluted in Antibody Diluent (S 0809, Dako Denmark), in proportion 1:1000 for CB1, 1:200 for CB2, 1:5000 for S100A6 and 1:50 for CacyBP/SIP. The procedure was followed by incubation with secondary antibody (conjugated to horseradish peroxidase-labelled polymer). The bound antibodies were visualised by 1 min incubation with liquid 3,3′-diaminobenzidine (DAB) substrate chromogen. The sections were finally counterstained in QS haematoxylin (H-3404, Vector Laboratories, Burlingame, CA, USA), mounted and evaluated under light microscopy. Appropriate washing with S3006 Wash Buffer (Dako Denmark) was performed between each step. Specificity tests performed for the CB1, CB2 S100A6 and CacyBP/SIP antibodies included negative control, where the antibodies were replaced by normal rabbit serum (Vector Laboratories, Burlingame, CA, USA) at respective dilution. For negative control, no immunostaining was observed in the heart tissues under the omission of the primary antibodies.

Histological preparations were subjected to a visual analysis using an Olympus BX41 light microscope with an Olympus DP12 digital camera and a PC computer and documented.

### Quantitative analysis

Images from five randomly selected microscopic fields, each field of 0.785 mm^2^, in magnification of 200x (20x the lens and 10x the eyepiece) from all heart sections were submitted for morphometric evaluation by using NIS Elements AR 3.10 Nikon software for microscopic image analysis.

The number of cardiac muscle cells were counted in each analysed image (only those cardiomyocytes with visible nucleus/nuclei; were taken into account); then the numbers cardiomyocyte cells were converted and presented as mean values per 1 mm^2^ section area.

In each analysed image of the heart, the width of 25 randomly selected cardiomyocytes was measured and presented as mean values.

The intensity of immunohistochemical reaction was measured, using a 0 to 256 grey scale level, where the completely *black pixels* were scored *0* and completely *white or bright* pixels were scored *256*.

### Real-time PCR

Total RNA was isolated using the High Pure FFPET RNA Isolation KIt (Roche). Quantification and quality control of total RNA was determined using the NanoDrop 2000 spectrophotometer (ThermoScientific, Waltham, MA, USA). An aliquot of 1 μg of total RNA was reverse transcribed into cDNA using iScript™ Advanced cDNA Synthesis Kit for RT-qPCR (BIO-RAD, Berkeley, California, USA). cDNA synthesis was performed in a final volume of 20 μl using a Thermal Cycler (Model SureCycler 8800, Aligent Technologies). For reverse transcription, the mixtures were incubated at 46 °C for 20 min then heated to 95 °C for 1 min and finally rapidly cooled at 4 °C.

Quantitative real-time PCR reactions were performed using the CFX96 Real-Time System (BIO-RAD, Berkeley, California, USA) with the SsoAdvanced™ Universal SYBER® Green Supermix (BIO-RAD, Berkeley, California, USA). Specific primers for the CNR1 (CB1), CNR2 (CB2), S100A6, CacyBP/SIP and GAPDH were designed by the BIO-RAD Company. The housekeeping gene GAPDH was used as the reference gene for quantification. In order to determine the amounts of tested genes expression levels, standard curves for each gene separately were constructed with serially diluted PCR products. PCR products were obtained by amplification of cDNA using specific primers as follows: CNR1 (qHsaCED0043777, BIO-RAD), CNR2 (qHsaCED0038847, BIO-RAD), S100A6 (qHsaCED0048256, BIO-RAD), CACY BP (qHsaCED0043669, BIO-RAD) and GAPDH (qHsaCED0038674, BIO-RAD). qRT-PCR was carried out in a dublete in a final volume of 20 μl under the following conditions: 2 min polymerase activation at 95 °C, 5 s denaturation at 95 °C, 30 s annealing at 60 °C for 35 cycles. PCR reactions were checked by including no-RT-controls, by omission of templates and by melting curve to ensure only a single product was amplified. The relative quantification of gene expression was determined by comparison of values of Ct using the ΔΔCt method. All results were normalised to GAPDH.

### Statistical analysis

All data were analysed for statistical significance using the software computer package Statistica Version 12.0. The mean values were computed automatically; significant differences were determined by two-way ANOVA test; *p* < 0.05 was accepted as significant**.**

## Results

Clinical baseline characteristics of the studied men and women, including the mean values of age, body weight and body mass index (BMI), are presented in Table 1. There were no differences in terms of average body weight and BMI between subjects older than 50 years and those under 50. Men were heavier and had higher BMI compared to women in both studied age groups (Table 1).

**Fig. 1:**
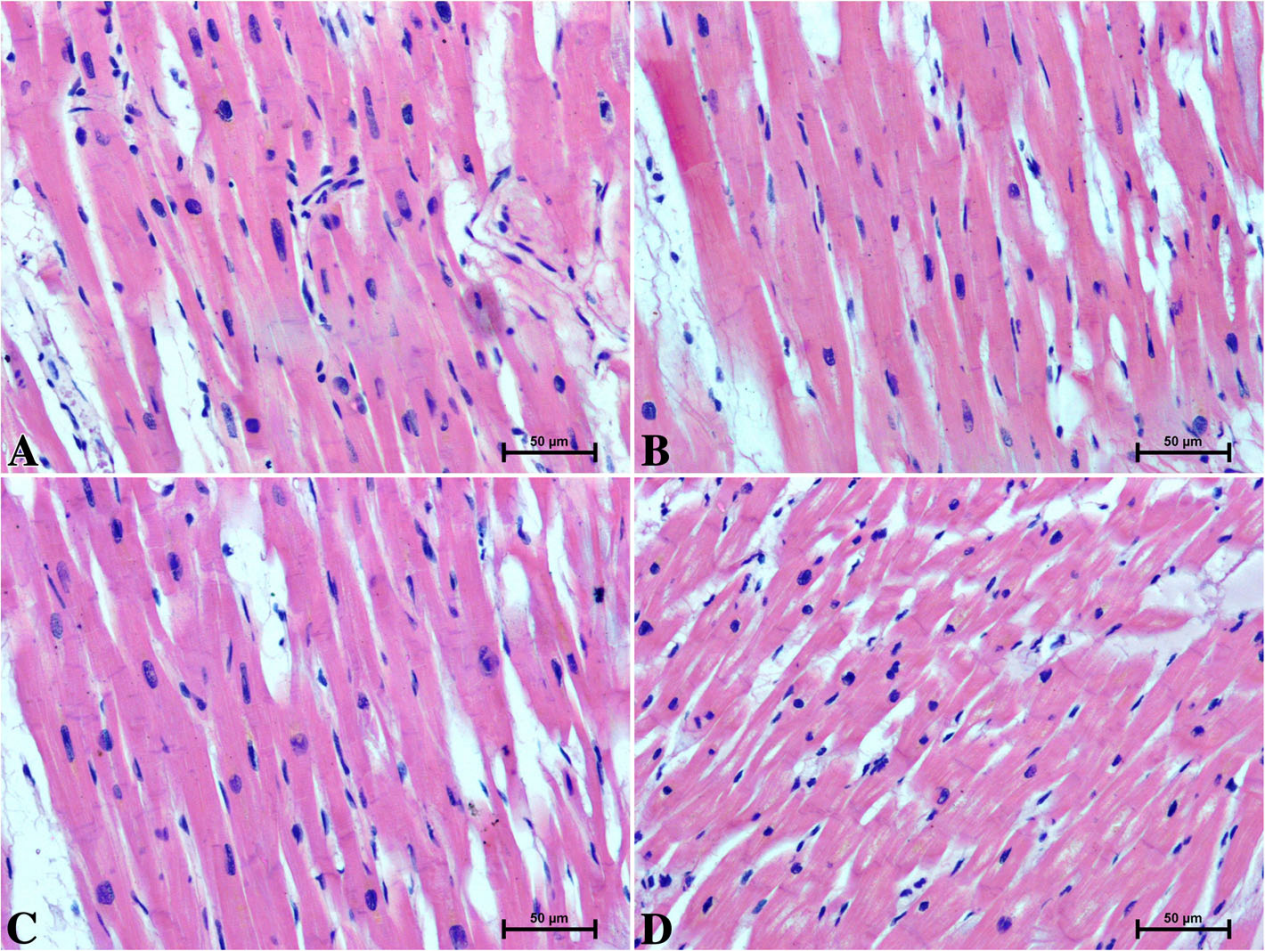
The heart section of patients under 50 years old **a** men **b** women and over 50 years old **c** men **d** women, H&E staining

Routine H + E staining showed no microscopic pathological changes in the hearts of men and women (Fig. 1).

**Fig. 2:**
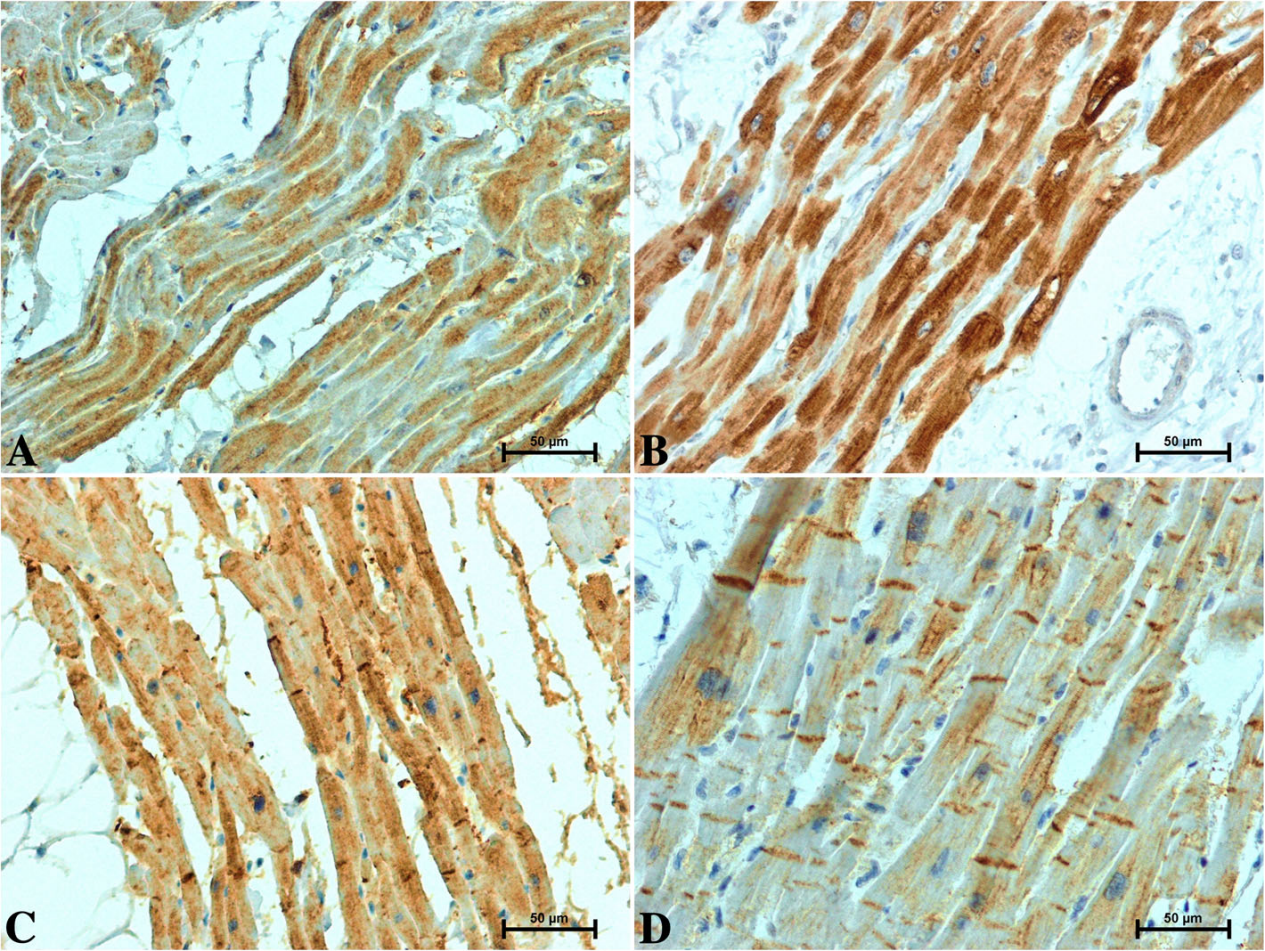
Representative images of CB1 immunolabelling in the heart of patients under 50 years old **a** men **b** women and over 50 years old **c** men **d** women

**Fig. 3:**
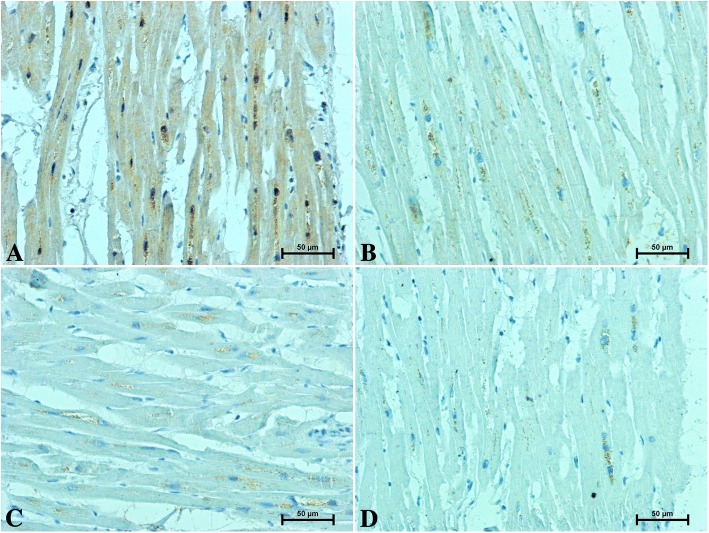
Immunolocalization of the CB2 receptor in the heart of patients under 50 years old **a** men **b** women and over 50 years old **c** men **d** women

**Fig. 4:**
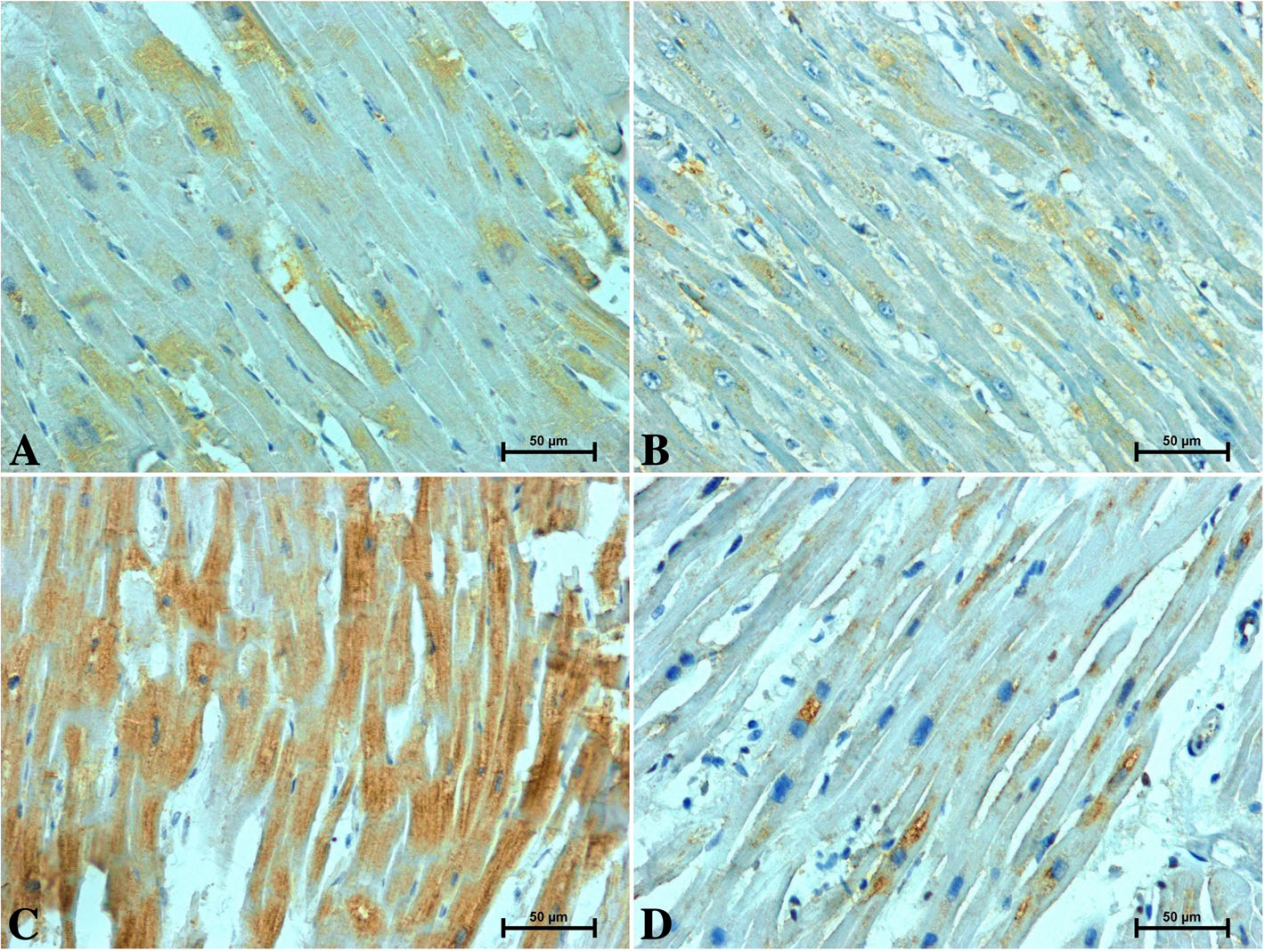
Immunohistochemical reaction determining S100A6 in the heart of patients under 50 years old **a** men **b** women and over 50 years old **c** men **d** women

**Fig. 5:**
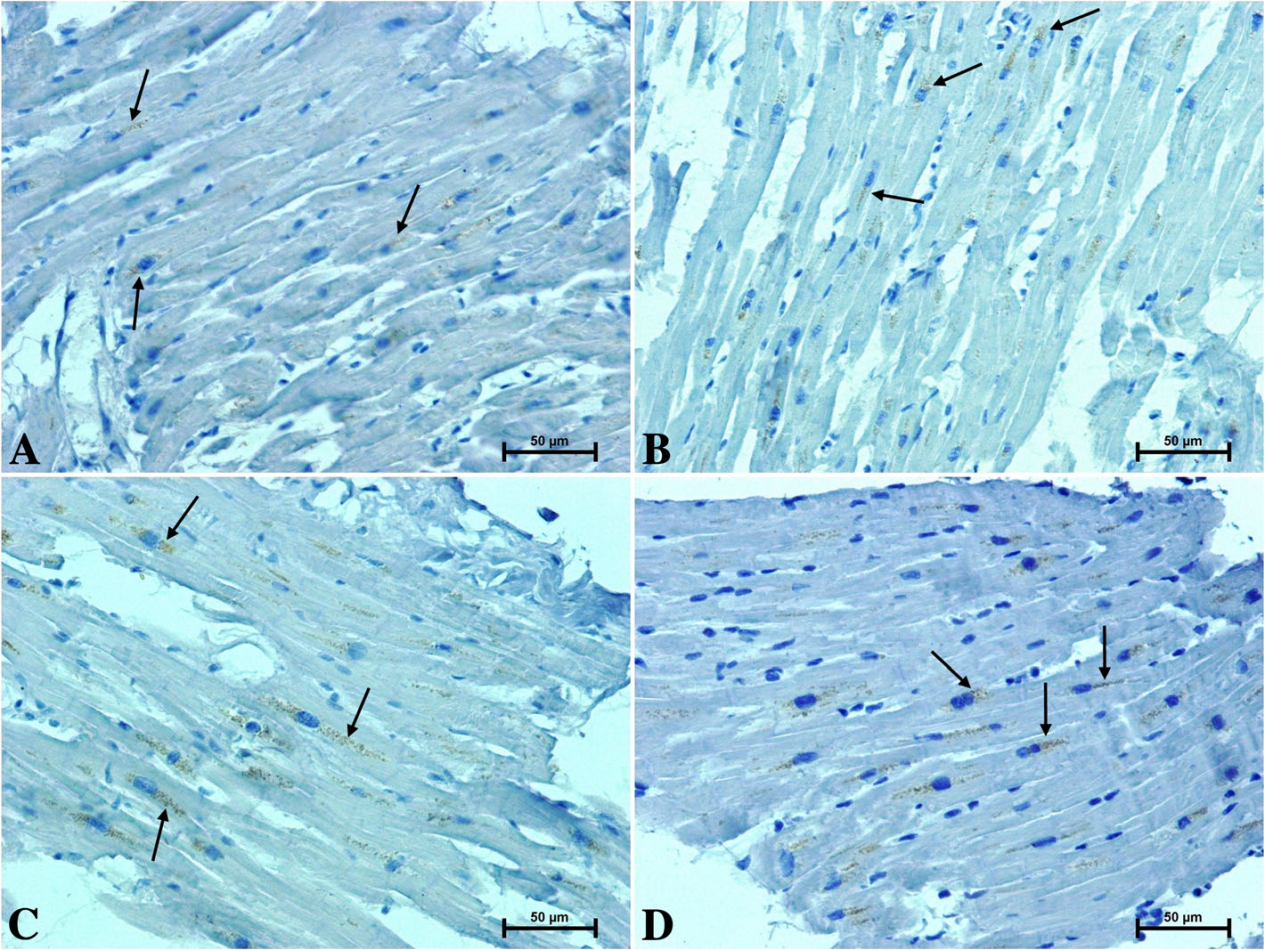
Positive CacyBP/SIP-immunostaining in heart of patients under 50 years old **a** men **b** women and over 50 years old **c** men **d** women

The performed immunohistochemical tests revealed a positive reaction of CB1, CB2 receptors, S100A6 and CacyBP/SIP in the hearts of all studied men and women, although the density and intensity of reactions varied between the sexes and age groups (Figs. 2, 3, 4 and 5).

Immunolabelling of the CB1 receptor in the hearts of subjects under 50 gave an intense reaction in the cytoplasm of cardiac cells (Fig. 2a, b) with noticeably weaker CB1-immunoreactivity in the heart of men (Fig. 2a) than in women (Fig. 2b). In the hearts of subjects over 50 (Fig. 2c, d), the intensity of cytoplasmic CB1-immunostaining in cardiomyocytes was considerably lower compared to those under 50 (Fig. 2a, b). The CB1-immunoreactivity in the hearts of men over 50 (Fig. 2c) was relatively greater compared to women of the same age (Fig. 2d). In the hearts of older men and women, strong CB1-immunoreaction in intercalated discs (ICDs) (Fig. 2 c, d) was also observed, which was not the case in individuals under 50 (Fig. 2a, b).

The CB2 receptor was identified in the hearts of men and women in the form of small brown-stained granules localised in the proximity of cardiomyocyte nuclei (Fig. 3a–d). In the hearts of men under 50 (Fig. 3a), CB2-immunostaining was stronger than in women belonging to the same age group (Fig. 3b). CB2-immunoreactivity in the hearts of older subjects (Fig. 3c, d) was significantly weaker compared to those under the age of 50 (Fig. 3a, b). The intensity of CB2 immunoreaction in the hearts of men over 50 (Fig. 3c) was weaker than in the age-matched women (Fig. 3d).

In the hearts of younger individuals, weak to moderate S100A6-immunoreactivity in the cardiomyocyte cytoplasm was noted (Fig. 4a, b). S100A6-immunostaining in the hearts of older subjects was significantly stronger compared to those under 50 years of age (Fig. 4c, d). In both studied age groups, the S100A6 immunoreactivity was higher in men hearts (Fig. 4 a, c) when compared to women (Fig. 4b, d).

Immunostaining of CacyBP/SIP in the hearts of men and women revealed its presence in the perinuclear area of cardiomyocytes (Fig. 5a–d). CacyBP/SIP-immunoreaction in the hearts of subjects under 50 was modest (Fig. 5a, b), whereas a noticeable more intense reaction against CacyBP was found in the myocardium of older individuals (Fig. 5c, d). There were no sex differences in the intensity of immunoreaction against CacyBP/SIP in hearts of subjects under 50 (Fig. 5a, b), although in men over 50 (Fig. 5c), CacyBP/SIP immunoreactivity was marginally weaker compared to the age-matched women (Fig. 5d).

Computer image analysis confirmed visually perceived age-related changes in the intensity of the immunohistochemical reaction against CB1, CB2, S100A6 and CacyBP/SIP in the hearts of men and women (Table 2).

**Table 2:** Intensity of immunoreaction against cannabinoid receptors (CB1, CB2), S100A6, CacyBP/SIP, in men and women hearts (scale from 0 (black pixel) to 256 (white pixel))

Group of patients		No. of measurements	Intensity of immunohistochemical reaction in the men heart				
			CB1		CB2	S100A6	CacyBP/SIP
			Cardiomyocyte cytoplasm	Intercalated discs	Cardiomyocyte cytoplasm	Cardiomyocyte cytoplasm	Cardiomyocyte cytoplasm
Under 50 years old	Men	2250	136.1 ± 1.76	ND	148.1 ± 1.75	162.0 ± 2.83	172.3 ± 1.90
	Women	1875	118.8 ± 2.87^♯^	ND	167.6 ± 2.64^♯^	177.3 ± 1.79^♯^	171.7 ± 2.89
			↑^♯^		↓^♯^	↓^♯^	
Over 50 years old	Men	2250	142.0 ± 1.72*	79.5 ± 3.32*	186.3 ± 2.91*	132.4 ± 2.56*	152.4 ± 1.85*
	Women	1875	155.6 ± 2.53*^♯^	86.9 ± 4.71*	177.5 ± 3.45*	150.1 ± 2.93*^♯^	147.0 ± 1.82*^♯^
			↓* ↓^♯^	↑*	↓* ↑^♯^	↑* ↓^♯^	↑* ↑^♯^

Data present mean ± SE

*p < 0.05 patients over 50 vs patients under 50 years old

^♯^*p* < 0.05 women vs men belonging to the same age group

^↓^Weaker immunohistochemical reaction

^↑^More intense immunohistochemical reaction

*ND* not detected

**Fig. 6:**
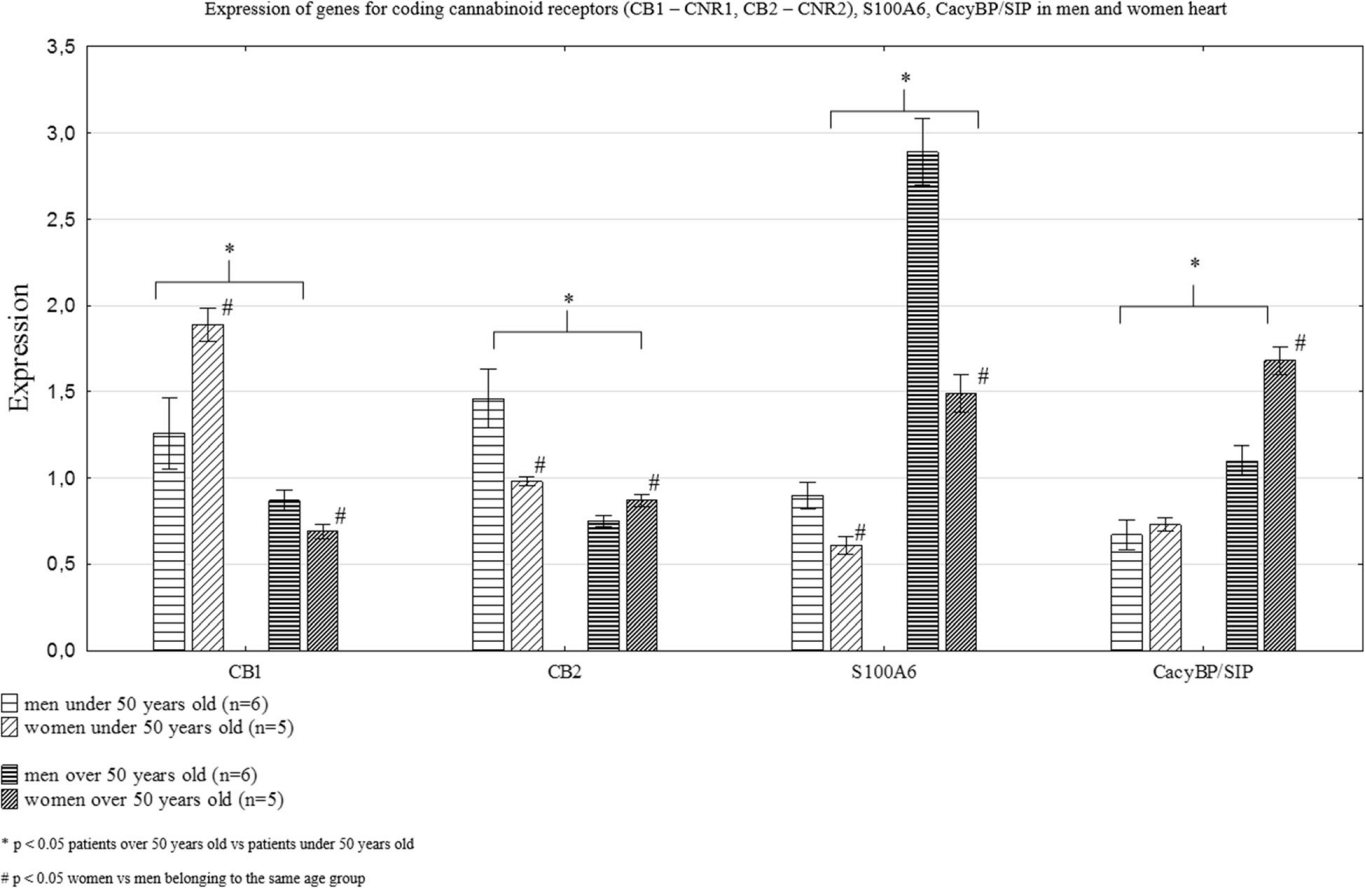
Expression of genes coding for cannabinoid receptors (CB1 and CB2), S100A6 and CacyBP/SIP in male and female hearts

The expression of genes coding CB1, CB2, S100A6 and CacyBP/SIP in the human heart was sex and age-dependent (Fig. 6, Table 3). The subjects over 50 years old had lower expression of genes coding CB1 and CB2 receptors, whereas intensified expression of S100A6 and CacyBP/SIP genes (Fig. 6, Table 3). The men under 50 years old had lower expression of CB1 gene and higher expression of CB2 gene compared to age-matched women, while in the group of subjects over 50 years old, the sex differences in expression of CB1 and CB2 genes were inverted (Fig. 6, Table 3). The expression of the S100A6 gene was intensified in men compared to women in both age groups (Fig. 6, Table 3). The men older than 50 years had lower expression of CacyBP/SIP gene compared to age-matched women, while in the group of subjects under 50 years old, there were no sex differences in expression of CacyBP/SIP gene (Fig. 6, Table 3).

**Table 3:** Expression of genes coding for cannabinoid receptors (CB1–CNR1, CB2–CNR2), S100A6 and CacyBP/SIP in male and female hearts

Group of patients			Expression of genes			
		No. of patients	CB1	CB2	S100A6	CacyBP/SIP
Under 50 years old	Overall	11	1.5 ± 0.50	1.2 ± 0.39	0.8 ± 0.21	0.7 ± 0.16
	Men	6	1.3 ± 0.50	1.5 ± 0.42	0.9 ± 0.18	0.7 ± 0.21
	Women	5	1.9 ± 0.20^♯^	1.0 ± 0.05^♯^	0.6 ± 0.11^♯^	0.7 ± 0.08
			↑^♯^	↓^♯^	↓^♯^	
Over 50 years old	Overall	11	0.8 ± 0.15*	0.8 ± 0.10*	2.2 ± 0.81*	1.4 ± 0.35*
	Men	6	0.9 ± 0.14*	0.7 ± 0.08*	2.9 ± 0.47*	1.1 ± 0.21*
	Women	5	0.7 ± 0.09*^♯^	0.9 ± 0.08*^♯^	1.5 ± 0.24*^♯^	1.7 ± 0.17*^♯^
			↓* ↓^♯^	↓* ↑^♯^	↑* ↓^♯^	↑* ↑^♯^

Data present mean ± SE

*p < 0.05 patients over 50 vs patients under 50 years old

^♯^*p* < 0.05 women vs men belonging to the same age group

The quantitative analysis revealed a decrease in the number of cardiac muscle cells in the heart of men over 50 years old compared to men under age of 50 (cardiomyocytes number per 1 mm^2^ section area was 60.9 ± 0.72 and 69.8 ± 1.22 respectively). In women, the number of cardiomyocytes remained unaffected by age (cardiomyocytes number per 1 mm^2^ section area was 69.4 ± 1.77 and 71.5 ± 1.21 for women over 50 and women under 50 respectively) (Table 4).

**Table 4:** The number of cardiac muscle cells per 1 mm^2^ section area and width of cardiomyocytes in men and women hearts

Group of patients		No. of measurements	The number of cardiac muscle cells per 1 mm^2^ section area	No. of measurements	Width of cardiomyocytes [μm]
Under 50 years old	Men	90	69.8 ± 1.22	2250	9.2 ± 0.33
	Women	75	71.5 ± 1.21	1875	9.6 ± 0.32
Over 50 years old	Men	90	60.9 ± 0.72 *	2250	11.1 ± 0.32 *
	Women	75	69.4 ± 1.77 ^♯^	1875	9.9 ± 0.34 ^♯^

Data present mean ± SE

*p < 0.05 patients over 50 vs patients under 50 years old

^♯^*p* < 0.05 women vs men belonging to the same age group

The performed morphometric analysis demonstrated cardiomyocyte hypertrophy in men older than 50 years as compared to younger men (cardiomyocytes width 11.1 ± 0.32 and 9.2 ± 0.33, respectively), whereas in women over 50, the width of cardiomyocytes was comparable with younger women (9.9 ± 0.34 and 9.6 ± 0.32 respectively) (Table 4).

## Discussion

Women live on average 4 to 6 years longer compared to men. The increased mortality among men is primarily associated with cardiovascular complications, which appear at an earlier age and progresses more rapidly than in women [21, 22, 27]. In studies concerning the effect of ageing on the human heart, Olivetti et al. [23] have found that in men, loss of cardiomyocytes occurs at the rate of 64 × 10^6^ cells per year of lifetime, while in women, the total number of myocardial cells remains essentially constant during their life span. Clinical studies on the hearts of subjects without a cardiovascular history and patients who suffered heart failure have demonstrated a higher index of cardiomyocyte apoptosis in men compared to women [28, 29]. Several experimental data have revealed that female rats suffer less pronounced cardiomyocyte injury after the administration of cardiotoxic doxorubicin, hydrogen peroxide or I/R period compared to males ones [30–32]. Therefore, based on the above statement, the existence of protective mechanisms preventing cardiac deterioration in women has been suggested, although they have not been fully elucidated yet.

In the current report, we present the first comparative evaluation of cannabinoid receptor (CB1 and CB2), S100A6 and CacyBP/SIP distribution and gene expression in the hearts of healthy individuals with regard to sex and age. Our study demonstrated a decreased density of both cannabinoid receptors in the cardiomyocyte cytoplasm in the hearts of older subjects. In the hearts of younger men, CB1-immunoreactivity was weaker and CB2-immunoreaction was stronger compared to women, but in older individuals, the reverse was observed—greater CB1-immunostaining and less intense CB2-immunoreactivity in men than in women. Noteworthy is the fact that in men and women over 50, the presence of the CB1 receptor was detected also in ICDs, which was not the case in younger subjects. The results of the present study also indicate an enhanced immunoreaction to S100A6 and CacyBP/SIP in the hearts of individuals over 50 years old compared to those under 50 years of age. We observed stronger S100A6-immunoreactivity in the hearts of men in both studied age groups. In younger individuals, there were no sex differences in the intensity of CacyBP/SIP immunoreactivity in the heart, while CacyBP/SIP immunostaining in the hearts of men over 50 was weaker compared to the age-matched women.

The presented report revealed also age and sex-related differences in the expression of CB1, CB2, S100A6 and CacyBP/SIP gene in the human heart, which was consistent with the immunohistochemically stated changes in peptide content. Oxidative stress is considered to be one of the main pathomechanisms implicated in the process of cellular ageing and apoptosis [33]. Cannabinoids significantly impact on oxidative balance in cardiac muscle cells [3, 4, 6, 7, 11]. Studies on rodent models of cardiomyopathy have demonstrated that CB1 receptor pathways promote oxidative damage in cardiac muscle cells, while the stimulation of the CB2 receptor limits oxidant-evoked injury in the myocardium [3, 4, 6, 7, 11]. The S100A6 and CacyBP/SIP proteins are implicated in cellular response to oxidative stress [34–36]. In vitro studies on various cell lines have demonstrated significant upregulation of S100A6 and CacyBP/SIP gene expression upon the induction of oxidative stress [34–36]. Literature data indicate higher susceptibility to oxidative stress and more severe oxidant-generated cardiomyocyte damage in males compared to females [31, 32]. The differences in CB1, CB2, S100A6 and CacyBP/SIP immunoreactivity and gene expression in the myocardium of men and women over the age of 50 years old observed in the current study might provide an explanation for sex differences in adaptation to oxidative stress in the ageing heart.

The key molecules implicated in the pathway of programmed cell death are caspases. Some recent evidence has revealed that cannabinoids and S100A6 impact on myocardial cell viability by modulating the caspase-dependent apoptotic pathway [3, 4, 8, 11, 15, 16]. Research on rodent models of cardiovascular disease has demonstrated that CB1-receptor pathways induce the activation of caspase and cardiomyocyte apoptosis, whereas the stimulation of CB2 receptors inhibits caspase expression and reduces cardiac cell death [3, 4, 8, 11]. Mofid et al. [15] and Tsoporis et al. [16] have found that S100A6 reduces caspase activity and increases the survival of cardiomyocytes subjected to hypoxia and the toxic action of TNF-α. Studies on rodents exposed to cardiotoxic conditions have revealed a greater increase in caspase activity in the cardiomyocytes of males compared to those of females [30, 31]. Therefore, the sex differences in CB1, CB2 and S100A6 gene expression and peptide content in the hearts of older individuals demonstrated in the present study might be associated with greater susceptibility of men to ageing-induced cardiomyocyte injury compared to women.

Mitochondria play a pivotal role in controlling the life and death of cells. The process of cell destruction is initiated by mitochondrial permeabilisation, which results in mitochondrial swelling and rupture. The release of the mitochondrial content results in the activation of proapoptotic intracellular enzymes and, subsequently, cell death [37]. Cannabinoids, acting via a CB2 receptor, influence the mitochondrial functionality and regulate the process of mitochondrial-dependent cell death [8, 9]. Report on rats subjected to I/R heart injury showed that treatment with CB2 receptors agonist prevent mitochondrial permeabilisation and following cardiomyocytes apoptosis [8, 9]. In a state of induced cardiomyopathy, rodent males display greater susceptibility to mitochondrial dysfunction in cardiomyocytes compared to females ones [30, 38, 39]. As demonstrated in our study, lowered CB2-immunoreactivity and expression of CB2 gene in the myocardium of men over 50 years old compared to age-matched women might provide a possible explanation for the more severe decline in mitochondrial function in the hearts of ageing men in comparison to women.

We stated in the current study that ageing leads to the redistribution of CB1 receptors to the region of ICDs in men’s and women’s hearts. Intercalated discs provide mechanical and electrical communication between cardiomyocytes, thus fulfilling a crucial role in maintaining the integrity of cardiac tissue and coordinating cardiac cell performance. ICDs structure contains adherens junctions, desmosomes and gap junctions and also a number of ion channels enabling the simultaneous spread of electrical potential across cardiac muscle cells. Voltage-dependent Ca^2+^ and Na^+^ channels are also among the ion channels identified in ICDs [40]. Experimental studies have demonstrated that cannabinoids, acting via CB1 receptors, modulate the activity of calcium and sodium voltage-gated channels [1]. Given the above statement, the observed displacement of the CB1 receptor to intercalated discs in the hearts of subjects over 50 years old might be associated with the role of CB1 receptors in the regulation of ion current through the channels occurring in ICDs. Several experimental data have indicated the disorganisation of ICD structure in the ageing heart [41]. The translocation of the CB1 receptor to ICDs observed in the hearts of older patients might be associated with age-induced ICD remodelling. However, further research should be performed to determine the effect of CB1 displacement on ICD function.

The results of the conducted quantitative and morphometric analysis revealed a reduction in the number of cardiac muscle cells and cardiomyocyte hypertrophy in men older than 50 years of age but no significant changes in the number and width of cardiac muscle cells in women belonging to the same age group. This observation is consistent with a report by Olivetti et al. [23] demonstrating a decrease in the number of cardiomyocytes and increase in myocyte cell volume in ageing men, but preserved the number of cardiomyocytes and the absence of myocyte cellular hypertrophy in the hearts of older women.

As demonstrated in the presented study and in the report by Olivetti et al. [23], ageing leads to successive myocyte cell loss in men; therefore, the remaining cardiomyocytes are subjected to greater stress. The hypertrophy of cardiac muscle cells observed in the hearts of older men might be associated with a compensative reaction for increased workload [23]. Cannabinoids and protein S100A6 perform a significant role in the process of cardiac hypertrophy [4, 11]. Research on murine models of cardiomyopathy has revealed that CB1-pathways promote a hypertrophic response from cardiomyocytes, whereas the stimulation of CB2 receptors is associated with the protection against cardiac hypertrophy. In vitro and in vivo studies have revealed that S100A6 reduces cardiomyocyte overgrowth induced by hypertrophic factors and ischemic heart injury [15, 17].

Considering the above statement, the sex disparities in CB1, CB2 and S100A6 expression and peptide distribution in the hearts of patients over 50 years old observed in the present study might constitute a possible mechanism explaining cardiomyocyte hypertrophy in older men and the preservation of myocardial cell size in ageing women.

## Conclusions

Obtained from each subject or from his/her family member, the immunohistochemical study results reported in the paper demonstrate the sex differences in the distribution of cannabinoid receptors (CB1, CB2), S100A6 and CacyBP/SIP proteins in the human heart as well as the alterations in the cannabinoid system and the studied proteins in the hearts of men and women over 50 years old.

By using Real-time PCR method, we revealed the age and sex-related differences in expression of CB1, CB2, S100A6 and CacyBP/SIP in the human heart, which was consistent with immunohistochemically stated changes in cardiac peptide content.

This report might contribute to a deeper understanding of the role of the cannabinoid system, S100A6 and CacyBP proteins in heart function as well as shed a new light on processes involved in age-related cardiac complications.

## Abbreviations

CacyBP/SIP: Calcyclin-binding protein/Siah-1-interacting protein
CB1, CB2: Cannabinoid receptors
MI: Myocardial infarction
TNF-α: Tumour necrosis factor-alpha
